# Utilization of ADCCs and quality of life among older adults: ethno-regional disparities in Israel

**DOI:** 10.1186/s12877-021-02674-0

**Published:** 2022-01-03

**Authors:** Adi Vitman-Schorr, Rabia Khalaila

**Affiliations:** 1 Shamir Research Institute, University of Haifa, Israel, 1290000, Kazrin, Israel; 2 Zefat Academic College, 11 Jerusalem St., P.O.B. 160, 13206 Zefat, Israel

**Keywords:** Day care center, Older adults, Quality of life, Ethnic groups

## Abstract

**Background:**

Adult day care centers (ADCCs) are a common service provided for frail older adults in the community. We examined the influence of older adults’ utilization of ADCC’s on their quality of life (QoL), and whether ethno-regional disparities are factors in the gaps found concerning QoL in different regions and between different ethnic groups.

**Methods:**

Cross sectional data were collected through structured interviews with 360 older adults attending ADCCs. Participants represented three ethnic groups and three regions in Israel. QoL was assessed by SF-36 questionnaire.

**Results:**

The results revealed a positive correlation between weekly hours at the ADCC, satisfaction with attending ADCC, and QoL. Older adults living in the central region had higher QoL than those living in the southern and northern regions. Veteran Israeli Jews reported higher QoL than FSU immigrants Israeli Arabs in all regions. Connection to one’s residential area was also correlated with QoL. A significant moderating effect of the interaction (ethnicity*area of residence) on QoL was also revealed.

**Conclusions:**

Attending ADCC is a vital community services to promote QoL in later life. Gaps in ADCC utilization between ethnic groups and residential region may cause disparities in QoL, specifically, in minority groups and those living in peripheral regions. Service providers should minimize the disparities by improving accessibility and availability for each person regardless of ethnicity and region of residence.

## Background

The burden of care for a community’s older adults falls primarily on family members [1–3], usually the children and spouse, if still living and, in some societies, a daughter in-law [4–6]. In Israel, great importance is placed on the ability of older adults to age at home. According to the “65+ Population in Israel” data, a very small percentage of older, veteran Jews (i.e., those who have lived in Israel for many years, whether born in Israel or arrived before 1990), former Soviet Union (FSU) immigrants (2.5%) and older Arabs (0.7%) live in a long-term institutional setting; most remain at home in the community [7]. To cope with the challenges of an aging population, the State of Israel, in an effort to promote keeping older adults in the community to age ‘in place’, supports them and their families with community-based services.

The current research relied on the active aging perspective, built on activity theory [8] and social integration theory [9], which posits that active engagement in social life promotes the older adult’s quality of life [10]. That engagement, in turn, protects the older adult from the threat of discrimination (mostly ageism). Research on activity theory and social integration theory also indicates the benefit of social involvement to the older adult. Self-esteem among the elderly is a notable dimension of well-being that benefits from activity, especially social activity [11]. These theories, however, neglect the possible influence of ethnicity and locale.

Adult day care centers (ADCCs), common service provided for frail older adults in the community, are financed by the National Insurance Institute’s Long-Term Care Insurance law (LTCI, 1988). The ADCCs supplement or partially replace the care provided primarily by the family legally responsible for their elderly members [12]. It is important to understand whether attending ADCCs influences QoL in later life, and whether ethno-regional disparities in utilizing these services can explain differences in QoL between sectors of a multi-cultural population, where most older adults live in the community.

## Literature review

### Community-based services for older adults and QoL

The formal social support system, consisting of a country’s health and social community- based services as the primary axis of care, has developed greatly in the last two decades in most developed countries, due to older adults’ desire to age in place [13]. Assistance for the elderly includes home health services, home delivered meals, transportation services, ‘panic buttons’, adults’ incontinence pads and ADCC services [14]. Research has suggested that the quantity and quality of both formal and informal social support affect older adults’ QoL and that core family members’ informal support results in a high level of satisfaction in older adults [15], At the same time, an increasing number of studies have explored the relationship between use of community services and QoL, demonstrating that frequent use of community services can effectively relieve the pressure on individuals and increase their subjective wellbeing and QoL [16].

ADCCs around the world are designed to provide the elderly with therapeutic social services and some health services. They facilitate interaction with a peer group, a source of emotional and health support for older adults. The intended goal of these community services is to promote home-living, delay nursing home placement, and maintain and restore cognitive and physical functioning [17, 18]. They also strive to improve psychosocial measures such as life satisfaction, QoL, interpersonal relationships, social activities and social integration [19]. ADCCs can be viewed as a community service with public health benefits for older adults [20].

ADCCs also provide a protected and stimulating environment to sustain the social, physical, and mental well-being of center-goers [21]. Social activities and social integration within the center are a remarkable credit to well-run centers [22]. The ADCCs in Israel also provide transportation to and from home, two meals daily, and personal care (health, hygiene, social work), health promotion, physical exercise, and laundry service [23]. For each participant, an individual care plan is prepared and provided for by a multidisciplinary team.

### Regional differences in using community-based services and QoL

The literature has revealed that QoL of older adults is related to their immediate living environment and the region in which they live [24–29]. There are two main aspects in the region of residence that can influence QoL: accessibility to services; and connection to the living environment. The literature shows that higher QoL in later life is related to both a deep connection to one’s living environment [24, 30–33] and to the extent and level of services provided as well as their level of accessibility by the individual [12, 24, 34]. A deep connection to the living environment is expressed by one’s close acquaintance with the physical environment together with strong feelings of belonging to a place and being part of its social and cultural fabric throughout the years [24]. Thus, it may be assumed that this sort of deep connection to the living environment leads to a high rate of utilization of community-based services by the older adult population [12].

The type of region of residence is another factor related to QoL among older adults. Urban regions are usually characterized by a good distribution of services and a variety of older adults’ services [35] as well as more accessibility possibilities, so that older adults’ services in an urban area are more likely to be utilized. This tendency toward utilization fosters social participation and high QoL [24, 36, 37]. In contrast, older adults living in rural areas, mostly found in peripheral regions, are typically offered fewer community services and less variety [35]; these living environments are also characterized by lower accessibility to services [38]. Older adults living in far-reaching, rural environments often report lower QoL than those older adults living in the urban-center regions [25].

### Ethnicity differences in using community-based serveries and QoL

Ethnic minorities often face greater difficulty accessing community-based services [39]. Service access problems of ethnic-minority older adults include language incompatibility [40], lack of transportation [41], inadequate knowledge of services [42], inadequate financial resources [39], institutional racism, mistrust of the system, lack of availability of culturally-sensitive services, personal beliefs, family dynamics, and culturally dissimilar styles of interaction between service users and providers [40, 42]. The result is that minorities often underuse community-based services, which might result in lower QoL than in the majority group.

There is a growing body of literature concerning racial/ethnic differences in QoL. Most of the studies have focused on Blacks and Hispanics and described poor QoL among these racial groups compared to Whites [43, 44]. Research in the US and the UK [45] has documented the perception of better QoL among members of the majority group and lesser QoL among those belonging to racial and ethnic minorities [46, 47]. Although minorities in Western countries are often socioeconomically disadvantaged, this is not always the cause of lower QoL [48]. Studies suggest that cultural norms and perceptions about ageing, as well as differing views of the role of older adults in society, also impact the perceived needs and expectations of older adults [49] and influence their QoL.

However, there are additional factors that may explain these differences. Ethnic minorities not only often live in regional peripheries, they also constitute the country’s social periphery even when living in urban areas, characterized by low socioeconomic status and education, and lower overall social conditions [25]. In Israel, the large ethnic group of FSU immigrants has reported a relatively low QoL compared to other immigrant groups such as Middle Eastern-North Africans, Eastern and Western Europeans, and Americans [21]. A study comparing older adults’ QoL in FSU immigrants vs. veteran Israeli Jews and Arabs found that the highest QoL was among veteran Israeli Jews, followed by FSU immigrants and Arabs [50]. Further examination found that the QoL of all groups declined over their lifetime, but the decreased QoL of the minorities (FSU and Arabs) was more pronounced [48].

### Interaction effect between area of residence and ethnicity on QoL

In many cases there is an overlap between belonging to an ethnic minority and living in the periphery. In Israel, most of the Arab population (65%) lives in the periphery regions (southern and northern Israel) [51]. The literature also raises the possibility of an interaction effect between area of ​​residence and ethnicity on older adult QoL. A British study found that QoL in minority groups was lower than that of white Britons, and that older adults living in the periphery reported lower QoL than older adults in the country’s urban and suburban center [25]. One study assessed the impact of ethnicity and place of residence on health status and their influence on QoL, the results showed that both variables were connected to QoL [52], suggesting on a possible moderation effect. Place of residence can also impact QoL through differences in income distribution, access to information and access to health care [53] including community-based services for older adults. It is, therefore, of great interest to measure the interaction of socially different groups and area of ​​residence in accordance to QoL.

In addition, socio-demographic characteristics such as age, marital status, education, gender, and income have been linked to QoL [21, 54]. The health-related QoL index measuring physical and mental functioning is often used as a reliable indicator of older adult QoL [54]. Poor physical health (chronic disease) and functional disability (ADL) are both associated with low QoL [21, 55–58], whereas good physical health is the leading predictor of a high level of physical and mental well-being [43].

The literature review shows that there are research studies concerning the QoL and how it is affected by community-based services; however, the connection between the way older adults use ADCCs (number of hours and utilization), satisfaction level from the ADCCs and QoL, has been overlooked. The current research aims to identify this aspect, and to understand the effect of ethno-regional disparities on using this service and on subsequent QoL.

## Method

### Study design

A cross-sectional study was used to test study objectives.

### Participants

The participants were recruited from 10 ADCCs from 10 different localities, selected randomly from a list of 25 districts from three regions in Israel. In compliance with sample size recommendations by Harrell, Lee and Mark [59], that is, 10 participants per parameter for multivariate analysis, we recruited 360 attendees for the current study using structured convenience sampling, representing three regions in Israel: center (180), southern periphery (90), and northern periphery (90). Three ethnic groups of 120 participants each were represented: veteran/native Israeli Jews, immigrants from the FSU (following collapse of the Soviet regime in 1991), and native Israeli Arabs. The quota of samples was determined according to the minimum sample size need for using a parametric statistical test for each category (> 30). Three ethnic groups live in each area; therefore, we need a total of at least 90 cases in each area and 90 cases of each ethnic group. Due to the disproportion of residents in each of the three areas in Israel (50% of the population in the central area, and about 25% each in the southern and northern areas), the sample in the central area was doubled.

Inclusion criteria were: age > 60; ability to speak Hebrew, Russian or Arabic; frail in terms of having physical difficulties in performing activities of daily living (ADL); and cognitively intact.

#### Ethical considerations

Institutional review board approval (IRB application number: REDACTED) was obtained prior to the study. All participants received an explanation of their right to withdraw at any time. Participants gave informed consent and they received a guarantee of confidentiality.

##### Data collection

Data were collected using a structured questionnaire administered in Hebrew, Arabic, and Russian after being independently translated by three bilingual speakers (Speakers of English and one of the three native languages); and validated in a pilot study of 10 respondents from each ethnic group (Jews, Arabs, and FSU immigrants (. Issues related to both the content and the clarity of the questionnaire were addressed prior to data collection. The data were collected from September 2016 to April 2017 in one-hour-long, face-to-face interviews conducted at the day care centers.

The interviewers arrived at the day care centers in coordination with center directors who agreed to allow participation in the study. The interviewers contacted visitors that day and asked for participation consent after providing an explanation and answering questions about the study and its purpose. Participants were interviewed on the days of data collection until the researchers reached the quota requested for each region and ethnic group. None of the 360 participants had missing data on variables of interest and all were included in the present analysis.

#### Measures

##### Dependent variable –quality of life

QoL was measured by a 36-Item Short-Form Health Survey (SF-36) [60] using a Likert-type scale 0–100 scoring system, originally designed as a generic indicator of health status for population surveys and evaluative studies of health policy [61]. The SF-36 has been widely used and translated, including into Hebrew and Arabic as one dimension [62, 63]. The scale was translated into Russian from English by two bilingual translators using the back-translation method, and validated in a pilot of 10 respondents from the FSU group. It comprises eight scales of a total of 36 items: physical functioning (10 items), role-physical (4 items), pain (2 items), general health perception (5 items), vitality (4 items), social functioning (2 items), role-emotional (3 items), mental health (5 items), and one question of comparative evaluation, comparing current health condition to health condition 1 year prior. Scores for the eight components range from 0 to 100, with higher scores indicating better QoL. The overall Cronbach’s α coefficient of the SF-36 questionnaire was 0.78, while the respective Cronbach’s α coefficients for each of the seven scales were > 0.70.

##### Independent variables– using community-based services

Three factors were used to assess utilization of community-based services. According to Israel’s Nursing Care Law, applicants eligible for LTCI benefits must be citizens, over the official age of retirement (64 for women and 67 for men), live in the community in their own home or in rental housing, and needing assistance to carry out daily activities (dressing, bathing, eating, walking indoors, etc.), or need constant supervision. Nursing benefit categories before November 2018 were divided into 3 levels, depending on the degree of dependence of the client on another person. Each level provides the elderly person with weekly care hours/ service units ranging from 5.0 h (level A) to 22 h (level C). The older adults can convert all or part of the hours/units he receives into visiting hours at the day center. A daily visit to the center equals about 2.45 weekly care hours / service units. Respondents were asked to indicate the number of weekly care hours/ service units allotted to them by the LTCI benefits; and the actual number of these LTCI weekly care hours/ service units they utilize in ADCC attendance. Respondents were also asked to rate their satisfaction with attending ADCC (To what extent are you satisfied with the visit to the day center?), with answers that included: 1 (=not at all satisfied), 4 (Slightly dissatisfied), 3 (Neutral), 4 (Slightly satisfied), or 5 (=very satisfied).

##### Covariates

The study controlled for six socioeconomic variables, two-regional related factors, and two health related factors. *Sociodemographic variables* included gender, age, marital status, number of children, years of education, ethnicity, and perceived economic status. Age, number of children and years of education were defined as continuous measures. Gender was a dichotomous measure (0 = male, 1 = female). Marital status was coded as with partner = 1 or without partner (single, widowed or divorced) =0. Ethnicity was defined as: Veteran Israeli Jews = 1, Immigrants from the FSU = 2, and Israeli Arabs = 3. Perceived economic status is measured on a discreet scale with five categories: 1 = very good, 2 = good, 3 = fair, 4 = poor, or 5 = very poor. Ethnicity served in the current study as a moderator.

*Regional-related factors* included two factors: area of residence (coded central = 1, northern = 2, southern = 3) and connection to residential area. Connection to residential area was evaluated by four items, with the respondents asked to rate their agreement regarding the following items: “Feeling part of this area,” “Vandalism/Crime is a big problem in this area,” “Area is kept very clean,” and “If I were in trouble, there are people in this area who would help me.” Likert-type scores included: 1 (strongly disagree), 2 (disagree), 3 (agree) or 4 (strongly agree) with higher scores indicating greater connection to residential area. Total scores ranged from 4 to 16. Internal consistency for the entire measure in the current analysis (Cronbach’s alpha) was ά = .70. Area of residence served in the current study as a moderator.

*Health-related factors* included two measures: physical health and functional disability. For *physical health*, respondents specified whether they were ever diagnosed with any of ten chronic diseases, including heart failure, hypertension, cerebral vascular disease, diabetes, hyperlipidemia, and chronic lung disease. This variable was calculated as the number of cited diseases, with its score ranging from 0 to 10. For functional disability, care-recipients rated their experienced difficulties in ADL using the ADL Scale developed by Katz et al. [64]. The ADL score ranges from 0 to 8, with a high score indicating very limited functioning. The Cronbach’s α coefficient of the ADL scale was 0.71.

##### Data analyses

Descriptive analyses for sociodemographic variables and QoL scores were performed using frequencies, percentages, means, and SDs. To preliminarily examine the associations between participants’ characteristics and QoL, univariate analyses including Pearson correlation coefficient, *t* test, and one-way analysis of variance (ANOVA) were conducted. To further investigate the potential factors of elderly persons’ QoL, a two-step weighted least squares regression analysis (‘Enter’ method) was performed with the QoL score as the dependent variable, and the covariates and the three measures of using community-based services as independent variables. In the first we analyzed the first step of the multiple linear regression which included three factors of using community-based services: the number of hours per week allotted for home care, number of hours per week utilized to visit the day care center and respondents’ satisfaction with ADCC use. The second step included the respondents’ sociodemographic factors followed by, health-related factors, and region-related factors. A univariate general linear model was then conducted to explore the effect of the interaction (area of residence*ethnicity) on QoL. Analysis was performed using SPSS package version 25.

## Results

The majority of participants were women, either unmarried or without a partner. The age range was 60–98 years (*Mean* = 79.5, *SD* = 6.8). On average, each respondent had 2.9 children, ranging widely between 1 to 10 children. Average education was 9.8 years (SD = 4.9). One-half of the respondents lived in the center of the country and one-quarter lived in each of the northern and southern peripheries. The average score of connection to residential area was medium with small deviation in this sample. One-third of the respondents belonged to each of the three ethnic groups: veteran Israeli Jews, FSU immigrants, and Israeli Arabs. The respondents reported, on average, 3.3 chronic diseases, and about half of the respondents reported being limited in at least three activities of daily life (Table 1).

**Table 1:** Descriptive statistics of the study variables (*N* = 360)

Covariates		***N*** (valid per cent)	***Mean*** (***S.D***)	Range
Gender	Female	214 (59.4)		
	Male	146 (40.6)		
Age			79.5 (6.8)	60–98
Education			9.8 (4.9)	0–22
Marital status	No partner	266 (74.1)		
	Has partner	93 (25.9)		
Number of children			2.9 (2.0)	1–10
ADL			4.0 (1.7)	0–8
Number of chronic diseases			3.3 (1.8)	0–10
Perceived economic status			2.42 (1.1)	1–5
Ethnicity	Veteran Jews	120 (33.3)		
	FSU immigrants	120 (33.3)		
	Israeli Arabs	120 (33.3)		
Area of residence	Northern	90 (25.0)		
	Central	180 (50.0)		
	Southern	90 (25.0)		
Connection to the living area			2.4 (0.5)	1–4
**Independent variables**				
Weekly hours for home care services			12.9 (4.3)	5–22
Weekly hours utilized for attending the ADCC			8.6 (3.3)	4–15
Satisfaction with the day care center services			3.9 (0.9)	1–5
**Dependent variable**				
Quality of life			41.6 (16.0)	11.1–83.4

*ADL* Activities of daily living

On average, each participant was granted 12.9 h per week (SD = 4.3) for home care services under the LTCI; of which they utilized 8.6 h weekly (SD = 3.3) for attending ADCCs. Additional analysis revealed that the Israeli Arab respondents were granted more weekly hours under the LTCI (*Mean* = 14.0, *SD* = 3.6) than veteran Israeli Jews (*Mean* = 12.5, *SD* = 3.7), and FSU immigrants (*Mean* = 12.4, *SD* = 5.1). However, the Arab respondents utilized the day care center services (*Mean* = 7.7, *SD* = 3.3) less than did FSU immigrants (*Mean* = 8.8, *SD* = 3.3) and veteran Israeli Jews (*Mean* = 9.3, *SD* = 3.1) (data not shown). In addition, the respondents reported high levels of satisfaction from attending and using the ADCC services, with an average score of 3.9 on a scale of 1 to 5. Average scores on the QoL measure ranged from 0 to 100 with a *Mean* of 41.6 (*SD* = 16), a relatively medium level with little deviation (Table 1).

Seven of the eleven covariates were related to QoL (Table 2). Older respondents and those with higher ADL limitations and more chronic diseases were more likely to report lower QoL. Participants who perceived their economic status as higher were more likely to report high QoL. A correlation was also found between the three factors of community-based services and QoL, and between ethnicity and area of residence with the dependent variable-QoL. Differences in QoL by ethnicity and by area of residence were also revealed. However, gender, years of education, marital status and number of children were not found to be associated with QoL.

**Table 2:** Pearson correlation coefficients between independent variables and Quality of Life (*N* = 360)

Independent variables	1	2	3	4	5	6	7	8	9	10	11	12	13	14	15	16
1.QOL	1.0															
2.Gender (1 = male)	.02	1.0														
3.Age	−.11*	−.08	1.0													
4.Education	.02	−.09	.04	1.0												
5.Marital status	−.01	.36***	−.25***	−.29***	1.0											
6.Number of children	.01	.15**	.01	−.36***	.23***	1.0										
7.ADL	−.09*	.05	−.01	.08	.02	−.07	1.0									
8.Number of chronic diseases	−.15**	.01	.02	−.07	.03	−.08	.43***	1.0								
9.Perceived economic status	.25***	−.06	.02	.11*	−.07	−.06	−.05	−.15**	1.0							
10.Ethnicity - FSU	−.02	−.15**	.19***	.55***	−.37***	−.36***	.01	.04	0.09	1.0						
11.Ethnicity - Jews	.23***	−.11*	.05	.19***	.04	−.12**	−.10*	−.24**	.36***	−.08	1.0					
12.Area - North	−.03	−.05	.11*	−.01	−.11*	.02	−.17**	.19**	−.12*	.01	0.01	1.0				
13.Area – South	−.11*	.15**	−.03	−.07	.20***	.12*	−.17**	−.05	−.18**	.01	.01	−.33***	1.0			
14.Connection to the living area	.43***	−.07	.12*	−.18**	.06	.15**	−.17**	−.11*	.20***	−.35***	.49***	.13*	.12*	1.0		
15.Weekly hours for home care services	.14**	.08	−.06	−.03	.05	.01	.60***	.38***	−.01	−.09	−.08	−.13**	−.13**	−.12*	1.0	
16.Weekly hours utilized for attending the ADCC	.16**	−.05	−.06	.05	−.08	−.04	.21**	.16*	.14**	.04	.14**	−.12*	−.12*	.06	.38***	1.0
17.Satisfaction with the day care center services	.25***	−.06	−.16**	.02	.03	.01	−.16**	−.01	.12*	−.07	.21***	.05	−.04	.21**	−.07	.11*

*ADL* Activities of daily living, Gender (1 = male; 0 = female), Marital status (1 = has partner; 0 = no partner), Ethnicity – FSU (FSU immigrants = 1; 0 = Israeli Arabs), Ethnicity – Veteran Jews (Veteran Jews = 1; 0 = Israeli Arabs), Northern area (1 = Northern area; 0 = central area), Southern area (1 = Southern area; 0 = central area)

*p < .05. **p < .01, ***p < .001

A weighted least squares regression analysis (‘Enter’ method) was carried out to detect the factors associated with QoL controlling for the covariates (Table 3). All independent variables were entered into the regression models in two steps: (1) the three factors of community – based services, and (2) the covariates. We entered 16 independent variables (included dummy variables) in this analysis. The final model identified eight significant factors associated with QoL with an adjusted *R*^*2*^ of .22 (*p* < 0.001). The effects of the factors of the community-based services were (Δ*R*^*2*^= =.10, *p* < 0.001), followed by effects of the covariates (Δ*R*^*2*^= =.12, *p* < 0.001).

**Table 3:** Summary of weighted least squares regression analysis for predicting QoL among users of day care centers (*N* = 360)

Variables	Model 1		Model 2	
	B	β	B	β
Weekly hours for home care services	−.63	−.19***	−.83	−.26***
Weekly hours utilized for attending the ADCC	1.11	.25***	.79	.18**
Satisfaction with the day care center services	2.87	.18***	1.95	.12*
Gender (RC = Female)			2.60	.08
Age			.06	.03
Education			−.10	−.03
Marital status (RC = no partner)			.34	.01
Number of children			−.45	−.05
ADL			.58	.07
Number of chronic diseases			−.90	−.11*
Perceived economic status			−.50	−.01
Ethnicity - FSU immigrants (*RC* = Arabs)			2.30	.07
Ethnicity - Veteran Jews (*RC* = Arabs)			−.12	−.01
Northern area (*RC* = central area)			−3.70	−.12*
Southern area (*RC* = Central area)			−7.04	−.23***
Connection to the residential area			9.69	.33***
***Adjusted R***^***2***^	0.10		0.22	
***F*** **for change in** ***R***^***2***^	11.92***		4.12***	

*ADL* Activities of daily living, *RC* reference category

*p < .05. **p < .01, ***p < .001

Looking closely at the final model, the results show a negative correlation between weekly hours for home care services and QoL, respondents allotting a higher number of weekly hours for home care services under the LTCI were more likely to report lower QoL. However, respondents who utilized greater weekly hours (out of the allotted hours by LTCI) for attendance at ADCCs, as well as those satisfied with the ADCCs, were more likely to report higher QoL. In addition, four covariates were related to QoL as identified earlier in the bivariate analyses: connection to residential area was positively associated with QoL. In addition, a greater number of chronic diseases was associated with lower QoL scores. Moreover, the results demonstrated ethno- and regional differences in QoL between older adults attending the ADCCs. Respondents living in the central area were more likely to report a higher QoL than their counterparts living in the southern and northern areas (peripheral districts). However, no differences were found between ethnic groups, nor was an association found between gender, age, marital status, number of children, ADL, perceived economic status and QOL.

**Fig. 1:**
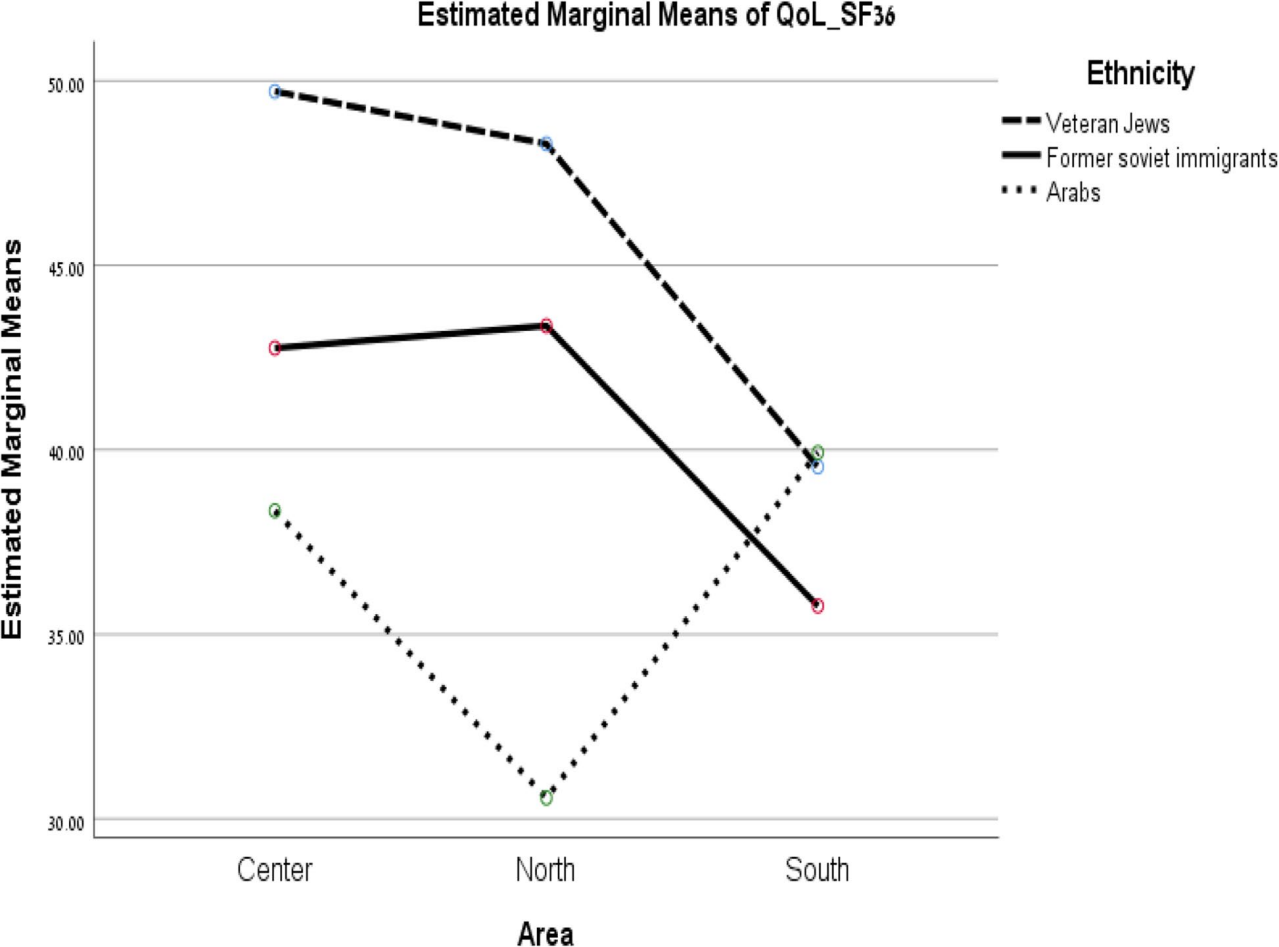
Relationship between area of residence and QoL at different ethnic groups, controlling for covariates

A univariate general linear model was next conducted to explore the effect of the interaction (area of residence*ethnicity) on QoL controlling for all other independent variables (Fig. 1). The interaction between area of residence–ethnicity was significant (*F = 3.45*, *p* = 0.008), suggesting that the effect of area of residence on QoL differed for each ethnic group. The significant interaction indicated that QoL of respondents living in the central region was higher for veteran Jews than for FSU immigrants and Israeli Arabs. In the northern periphery, QoL was higher for veteran Jews and FSU immigrants than for Israeli Arabs, whereas QoL in the southern periphery was higher for veteran Jews and Arabs than for FSU immigrants.

## Discussion

The current study examined the association between using community-based services and QoL among older adults, as well as the moderating effect of (ethnicity*area of residence) on QoL controlling for covariates. The results revealed that a higher-level QoL of older adults is related to more hours per week spent at an ADCC, and with greater satisfaction from attending and using ADCC services. However, a higher number of weekly hours for home care services allotted under LTCI was inversely correlated with QoL.

The correlation between more weekly hours spent at ADCCs and high satisfaction from ADCC use with higher QoL are consistent with prior studies [14, 65–67], which found that use of ADCCs can improve older adults’ QoL and enable them to age in place, in their familiar environment and community. These results can be explained in part by the services provided at the ADCCs, such as personal care, social activities, hairdressing, health promotion etc. Providing a wide variety of services responds to attendees’ different needs. It is worth noting, however, that the majority of attendees visit the ADCC in order to take part in the social activities [68], increasing both their satisfaction from the ADCC and their QoL [69, 70].

The negative link between weekly hours allotted under the LTCI for home care services and QoL may be explained through additional factors investigated in the current study, namely chronic disease and physical dysfunction. According to the LTCI criteria, older adults with poor physical health and a higher level of functional disability receive a higher number of weekly hours for home services; our results also showed a positive correlation between the number of chronic diseases and ADL disability and the number of weekly hours for home services. Indeed, the findings of previous research studies have shown that poor physical health and functional ability are related to poor QoL among older adults [21, 55–58].

The current study also highlights ethno-regional disparities in the standard of community services such as ADCCs provided to older adults, that can lead to differences in QoL among older adults. The results revealed differences in QoL between older adults from different ethnic groups and those living in different regions. QoL was higher for older adults living in the central region than for those living in the southern and northern regions. It was also found in the bivariate analysis that the QoL of the majority group, veteran Jews, was higher than that of minority group Israeli Arabs, but was not different than that of FSU immigrants. However, these ethnic differences were not found to be significant in the linear regression analysis. These findings concerning ethno- and regional disparities are consistent with prior studies conducted in other countries [25, 60, 68]. One explanation for this gap in QoL between regions may be related to the lower connection to the living environment among those living in the southern and northern areas compared with those living in the central area, as we found in our study. Our results showed that a lower connection to the living environment was associated with lower QoL. Further explanation for the disparities in QoL between ethnic groups, may also be related to the lower economic status of the older Arab citizens and FSU immigrants compared to the older Jewish citizens, as emerged in the current study. Indeed, previous studies [21], and our results showed that lower perceived economic status was correlated with lower QoL.

In Israel, ethno-regional disparities stem from the relationship of living environment and regional area to the quality of ADCCs, and therefore, to the experience of the older adults [71]. In most cases, minorities (that is, Israel Arabs and FSU immigrants) live in the periphery, where there are usually fewer services and, in many cases, the standard of services is much lower than in the central region, contributing to lower QoL in the peripheral regions. This situation may characterize similar situations for minorities in other countries (e.g., Afro-American and Latino communities in the US) who often suffer from a lower standard of health services [44], community-based services [72], and educational services [44], stemming from historical domination, resource inequity and stigma, as well as other reasons [73]. These gaps in the level, amount, dispersion, and accessibility of community services between majority and minority groups create a social periphery. Even when social and regional peripheries do not merge, one of these is sufficient to produce low QoL for ethnic minorities and other peripheral residents.

The ethno-regional disparities in QoL in our study are both demonstrated and can be explained by the interaction between ethnicity and area of residence. Our findings indicated that Arabs in the central and northern areas are the most underserved population, and as a result, have the lowest QoL. In the south, the FSU immigrants take their place as the population with the lowest QoL. The interaction model supports the conclusion that QoL is associated with ethnicity and regional location (variables taken separately and combined). Ethnic minorities are the most vulnerable groups (with Arabs more vulnerable than FSU immigrants) and while this is true for minorities living in the center, living in the periphery is an additional risk factor for low QoL.

Interestingly, the results showed that although Arab older adults received more hours for home care services under the LTCI, they utilized the ADCCs less than did Jews and FSU immigrants. One explanation for this gap may be low accessibility and availability in their living environment due to lack of infrastructure, incorrect choice of location and/or dispersion, lack of transportation, lack of ADCCs in the living area, and lack of information about the community services for older adults. The disparity may also be due to cultural issues such as stigma and negative views regarding the use of formal services for older adults in Arab-Israeli society [74], or because of the low preference of Arab family members to utilize the benefit of weekly hours allotted under LTCI on ADCCs and higher preference to utilize those hours on basic home care [4].

To summarize, the present study’s findings indicated the importance of community services (such as ADDCs) to maintain aging in place and QoL among older adults. The study also revealed the gaps in QoL between different ethnic groups and regions. It appears that minorities are at the highest risk for low QoL and that living in the regional and social periphery increases that risk.

### Implications

Policymakers and service planners should act on two different aspects. First, they need to establish more community services and social-oriented programs for the elderly population, particularly for disadvantaged populations such as minority groups and those living in the geographical periphery. The second aspect concerns the community services, which should be improved with an emphasis on accessible and quality services for older adults, mainly in the periphery and among the minorities. The day care center services should focus on physical accessibility, better dispersion, and transportation, as well as on improving the standard of services. Effort should be made by local social services to raise awareness and convince the Arab population regarding the importance of using community services in improving QoL. The LTCI should also “spread the news” regarding the older adults’ rights and the benefits older adults can achieve from taking advantage of ADCCs’ services.

The current study has two main limitations. One is the cross-sectional study design, which does not enable prediction of a causal relationship between variables. A future study should use longitudinal data to examine the relationship between using community-based services and QoL of older adults. A further limitation is the non-random selection of the convenience sample and lack of control group of individuals legally entitled to LTCI benefits who do not attend an ADCC at all, which limit generalizability. Despite these limitations, the present study represented different regions and the main ethnic groups in Israel, and provides initial insights into the mechanisms of the associations between using community services (ADCCs) and QoL among older adults in an ethno-regional disparities’ context, an important aspect in delivering community services to older adults that has not been widely studied thus far.

## Data Availability

The datasets generated and/or analysed during the current study are not publicly available due to third party restrictions, but are available from the corresponding author on reasonable request.
